# Source-free domain transfer algorithm with reduced style sensitivity for medical image segmentation

**DOI:** 10.1371/journal.pone.0309118

**Published:** 2024-12-27

**Authors:** Jian Lin, Xiaomin Yu, Zhengxian Wang, Chaoqiong Ma

**Affiliations:** 1 Sichuan Academy of Medical Science and Sichuan Provincial People’s Hospital, Chengdu, China; 2 School of Electronic Engineering, Chengdu University of Information Technology, Chengdu, China; University of Florida, UNITED STATES OF AMERICA

## Abstract

In unsupervised transfer learning for medical image segmentation, where existing algorithms face the challenge of error propagation due to inaccessible source domain data. In response to this scenario, source-free domain transfer algorithm with reduced style sensitivity (SFDT-RSS) is designed. SFDT-RSS initially pre-trains the source domain model by using the generalization strategy and subsequently adapts the pre-trained model to target domain without accessing source data. Then, SFDT-RSS conducts interpatch style transfer (ISS) strategy, based on self-training with Transformer architecture, to minimize the pre-trained model’s style sensitivity, enhancing its generalization capability and reducing reliance on a single image style. Simultaneously, the global perception ability of the Transformer architecture enhances semantic representation to improve style generalization effectiveness. In the domain transfer phase, the proposed algorithm utilizes a model-agnostic adaptive confidence regulation (ACR) loss to adjust the source model. Experimental results on five publicly available datasets for unsupervised cross-domain organ segmentation demonstrate that compared to existing algorithms, SFDT-RSS achieves segmentation accuracy improvements of 2.83%, 2.64%, 3.21%, 3.01%, and 3.32% respectively.

## 1. Introduction

With the increasing stringency of privacy protection policies, pathology data of patients tends to be monopolized by large institutions such as hospitals and remains inaccessible to external parties [1]. Simultaneously, the large volume of medical image data results in significant resource consumption during storage, transmission, and data loading processes. These constraints make accessing medical image data increasingly challenging in practical healthcare scenarios [2,3]. Unsupervised transfer learning has gained favor among scholars due to its outstanding knowledge transfer capabilities and has found extensive applications in medical image segmentation to address inter-domain distribution differences. However, such algorithms often presume continuous access to source domain data throughout the entire model training process [4,5]. In actual scenarios, the inaccessibility of source domain data makes these methods difficult to apply.

**Fig 1:**
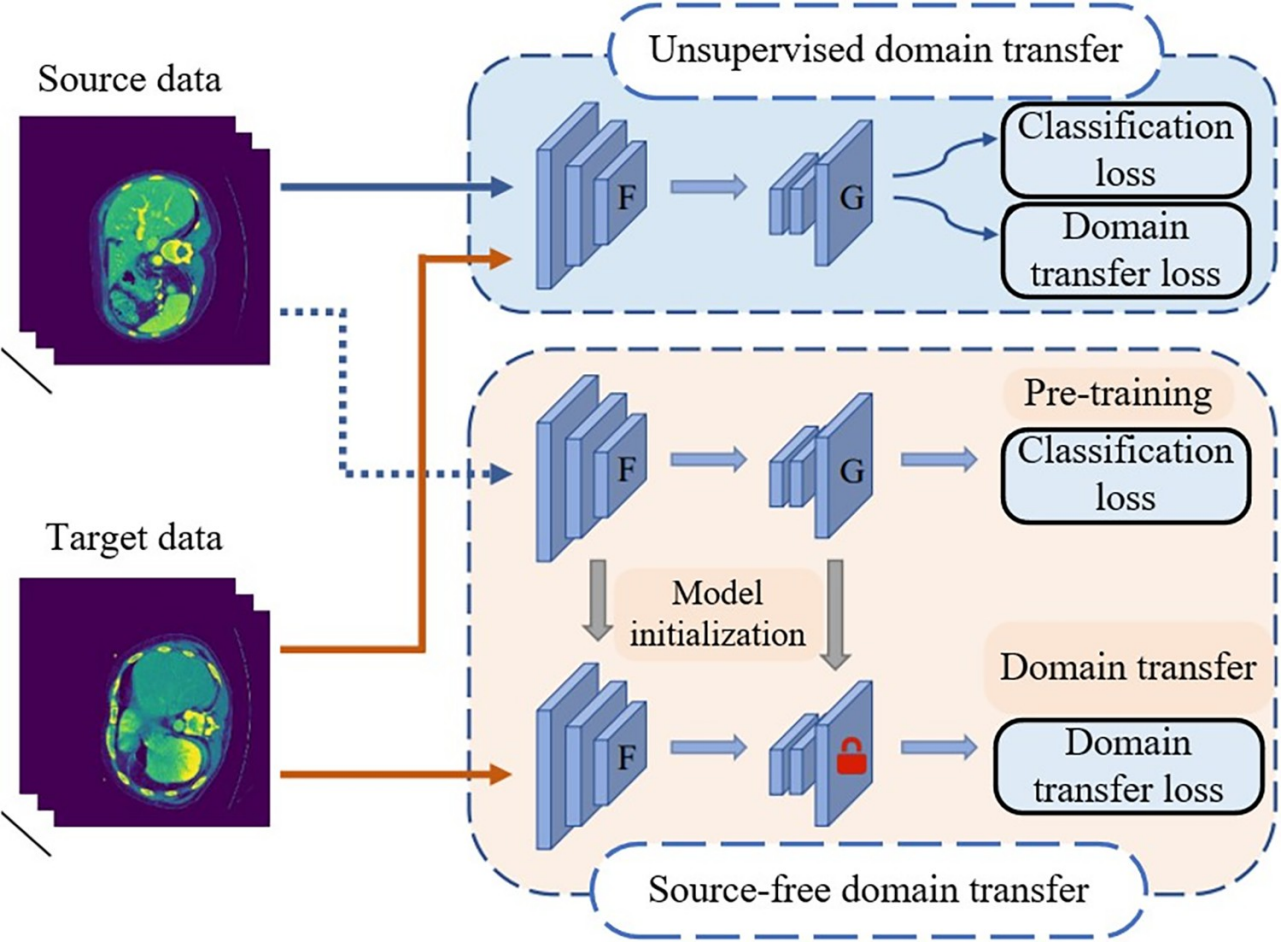
Comparison of unsupervised domain transfer and source-free domain transfer.

Currently, some scholars have conducted research on knowledge transfer with no using data, known as source-free domain transfer problem [6]. Fig 1 illustrates the difference between traditional transfer idea and source-free domain transfer idea. Traditional unsupervised transfer leaning requires simultaneous availability of both source and target data and achieve inter-domain knowledge transfer through continuous optimization of the objective function. In contrast, source-free domain transfer methods first pre-train the segmentation model with data and subsequently rely solely utilize this model and target domain data to achieve knowledge transfer. Since the source domain data cannot be accessed, this implies that knowledge transfer across domains must be achieved indirectly, solely utilizing the obtained information [7].

Current source-free domain transfer algorithms are divided to two types [8]. The first type relies on distribution simulation techniques, which replicate the distribution of source data by leveraging the source model and target data, thus compensating for the unavailability of source data. The second type employs statistical normalization techniques, which adjust the model automatically by altering the parameters of the batch normalization layer within the convolutional model.

Typically, information in medical images can be categorized into content information and style information. Content information represents the semantic content of the images, providing clues for knowledge transfer, while style information contributes to inter-domain distribution differences [9]. In scenarios without considering cross-data modalities, for different medical images of the same body part of a patient, their semantic information remains consistent, reflecting similar pathological information. Therefore, in cross-domain medical image segmentation, research is conducted along the lines of "transferring content information while avoiding style differences." For instance, conventional transfer learning approaches mitigate inter-domain stylistic discrepancies by employing methods like style conversion. This technique aligns target images with the style of source images, thereby alleviating domain disparities [10,11]. Nonetheless, current source-free domain transfer algorithms concentrate exclusively on leveraging the posterior distribution information. However, when confronted with cross-domain stylistic variations, these source-free domain transfer models experience a notable degradation in segmentation accuracy. Due to substantial style discrepancies between different domains, applying existing source-free transfer strategies to source models results in a continuous decline in segmentation accuracy during domain transfer, ultimately leading to irregular segmentation outcomes. This is because existing source-free transfer strategies, in scenarios where source domain data is inaccessible, cannot address style shifts as unsupervised transfer methods do. Instead, they solely focus on transferring content information, thereby confounding content and style information during domain transfer. Additionally, since existing source-free strategies rely on the initial predictions of the model for updates, significant initial prediction biases make it challenging for the model to update in the correct direction, trapping existing source-free strategies in the "error diffusion" dilemma. Therefore, addressing style shift issues in source-free scenarios holds significant research value [12,13].

To this end, we develop the SFDT-RSS to mitigate the impact of style shifts. SFDT-RSS initially trains the source model using a generalization approach, and subsequently adjusts the source model to the target without using source data. Then, SFDT-RSS conducts ISS strategy, based on self-training with Transformer architecture, to minimize the pre-trained model’s style sensitivity, enhancing its generalization capability and reducing reliance on a single image style. Simultaneously, the global perception ability of the Transformer architecture enhances semantic representation to improve style generalization effectiveness. In the domain transfer phase, the proposed algorithm utilizes a model-agnostic adaptive confidence regulation ACR loss to adjust the source model. Based on this, SFDT-RSS demonstrates more effective completion of source-free cross-domain medical image segmentation tasks.

The organization of this work is, Section 2 illustrates a review of the research status. Section 3 describes the design details. Section 4 conducts a comprehensive experimental evaluation. Section 5 concludes the work.

## 2. Related works

### 2.1 Unsupervised domain transfer algorithms

Contemporary unsupervised domain transfer algorithms for medical image segmentation commonly align the data distributions across domains within the feature space. Based on the implementation strategies, these algorithms can be categorized into instance-based weighting and representation learning methods. Opbroek et al. [14] proposed the reweighted support vector machine (RSVM) for lesion segmentation. RSVM iteratively updates the model, where the weights of misclassified samples are reduced to minimize their influence on model updates. Perone et al. [15] introduced a sample selection algorithm based on Knowledge Distillation for spinal cord gray matter segmentation, which utilizes exponential moving average strategy for parameter updating. During training, samples with high prediction consistency have a stronger impact on model updates. Instance-based weighting methods can demonstrate good performance under certain conditions. However, variations in the sample weight from different classes in the dataset may disrupt the balance of classes, altering the prior distribution information of the source domain, causing the classifier to consistently favor classes with higher total weights, thereby neglecting classes with lower weights.

Wang et al. [16] introduced an adversarial learning approach driven by boundaries and entropy for retinal image segmentation, generating more accurate boundary segmentation predictions similar to the source domain. Vu et al. [17] introduced the adversarial entropy minimization segmentation algorithm, which addresses distribution shift issues through pixel-level adversarial learning and entropy minimization. Zou et al. [18] studied the enhancement of confidence in pseudo-labels for the target domain through a self-supervised mechanism. They iteratively improved the confidence of pseudo-labels and retrained the model using these updated pseudo-labels in each iteration, thereby enhancing the adaptation of the target model. However, representation learning approaches assume that each sample in the source domain contributes equally to knowledge transfer. In practical data collection processes, random noise effects may cause samples to deviate extremely from the center of the data distribution in the source domain, treating these samples the same as other samples may lead to severe negative transfer, resulting in a decrease in model segmentation accuracy [18].

### 2.2 Source-free domain transfer algorithms

In real-world scenarios, the assumption that source domain medical image data is readily available is often invalid due to concerns regarding patient data privacy. As a result, traditional transfer algorithms become impractical. The concept of source-free domain transfer has garnered growing interest because it doesn’t rely on access to source data [19]. Examining existing approaches to source-free domain transfer for medical image segmentation, these methods can be broadly classified into two categories: those centered on simulating source domain distributions and those focused on statistically regularizing model parameters. Chen et al. [20] developed the denoised pseudo-labeling (DPL) source-free algorithm for retinal fundus image segmentation based on uncertainty and prototype estimation. DPL estimates pixel-level prediction uncertainty based on the self-supervised paradigm, identifies pseudo-labels with high uncertainty, and estimates category prototypes driven by prototype networks, calculating relative feature distances to pseudo-labels far from their corresponding class prototypes. Bateson et al. [21] utilizes inter-class proportion predictors to obtain posterior information of the source domain, minimizing unlabeled entropy loss defined on target domain data, and integrating it into the overall optimization objective of the model in the form of KL divergence. These methods tend to perform well when there is small distributional discrepancy. However, when the distributional discrepancy is significant, large errors may occur in both pseudo-labels and generated data, resulting in the problem of error diffusion and continuous decrease in model segmentation accuracy during training.

Statistical regularization-based source-free domain transfer methods adopt the opposite approach. These approaches adjust the parameters of batch normalization layers in convolutional models to align the target domain features with those of the source domain, thereby regularizing the target domain features. Liu et al. [22] proposed source-free transfer algorithm by using Adaptive Batch Statistics, which normalizes features of brain MRI images through low-order and high-order statistics. The algorithm highlights that imposing identical mean and variance across domains results in a reduction in model expressive capacity. Methods based on statistical regularization of model parameters require the use of convolutional neural network structures. Similarly, these methods may also lead to the problem of error diffusion.

## 3. Source-free domain transfer algorithm

### 3.1 Algorithm overview

**Fig 2:**
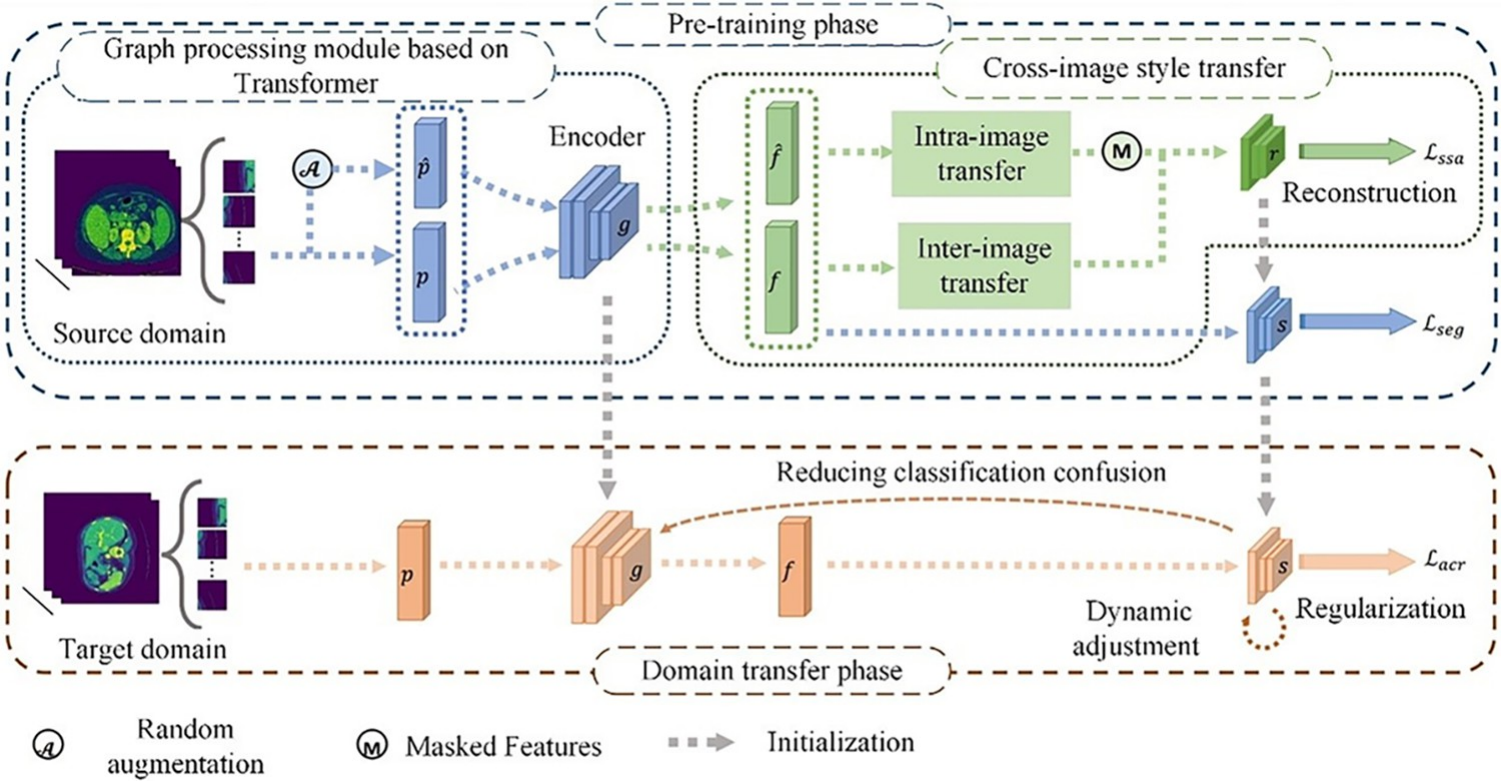
Medical image segmentation workflow of the proposed algorithm SFDT-RSS.

Many symbols and abbreviations are used in this paper, which is presented in Table 1. The proposed framework for medical image segmentation is illustrated in Fig 2. Compared to existing source-free domain transfer algorithms, this algorithm focuses on mitigating the adverse impact of style variations to prevent error diffusion issues. SFDT-RSS adopts a strategy of generalization before transfer. Initially, it mitigates the source domain model’s sensitivity to style variations through the ISS mechanism, thus improving the model’s generalization capability and reducing its dependence on a singular style. Subsequently, in the absence of source data, the algorithm employs the ACR loss to gauge pixel correlation, adjusting the source model to the target by heightening classification certainty. This approach further mitigates the risk of misclassification stemming from style discrepancies, ultimately facilitating knowledge transfer in a source-free context.

**Table 1:** Symbols and abbreviations in this paper.

Symbol	Meaning	Abbreviation	Meaning
$p_{k}^{n}$	*k*_th_ patch partitioned from image	SFDT-RSS	Source-free domain transfer algorithm with reduced style sensitivity
$A_{rand}(\cdot )$	Stochastic boosting operator	ISS	Interpatch style transfer
*P* ^*n*^	Raw patchset	ACR	Adaptive confidence regulation
${\hat{P}}^{n}$	Augmentation patchset	ASD	Average Surface Distance
*f* ^*n*^	Encoded features of raw patchset	RM	Random mask
${\hat{f}}^{n}$	Encoded features of augmentation patchset	CFP	Color fundus photographs

Here, the problem is defined. Firstly, a source domain model M_s_:X_s_→Y_s_ is pre-trained to classify each pixel of medical images. When transfer, source domain X_s_∈ℝ^H×W×1^ and its corresponding labels Y_s_∈ℝ^H×W×C^ are not available. The research objective of the proposed algorithm is to accurately identify the unknown target domain X_t_∈ℝ^H×W×1^ without accessing domains X_s_ and Y_s_.

### 3.2 Interpatch style transfer strategy

#### 3.2.1 Intra-image transfer

Prior to network input, medical images are divided into *K* equally-sized and non-overlapping image patches using the Transformer’s patch processing approach. These patches serve as the network’s input. Here, $p_{k}^{n}\in P^{n}$ represents the *k*_th_ patch partitioned from image x_n_, and $A_{rand}(\cdot )$ denotes a stochastic boosting operator. Stochastic boosting is applied to each patch contained in the image to obtain an augmented patch set, as shown below:

$${\hat{P}}^{n}=\cup_{k=1}^{K}A_{rand}(p_{k}^{n})$$ (1)

Then, raw patchset *P*^*n*^ and augmentation patchset ${\hat{P}}^{n}$ are input to network. The encoder *g* extracts feature from the input patches, denoted as $f^{n}=[f_{1}^{n},f_{2}^{n},\cdots ,f_{K}^{n}]$ and ${\hat{f}}^{n}=[{\hat{f}}_{1}^{n},{\hat{f}}_{2}^{n},\cdots ,{\hat{f}}_{K}^{n}]$, respectively. The following objective function is used as a constraint, to guarantee consistent feature distillation:

$${ℒ}_{inv}(\theta_{g})=\mathrm{argmin}\frac{1}{NK}\sum_{n=1}^{N}\sum_{k=1}^{K}\|f_{k}^{n}-{\hat{f}}_{k}^{n}\|+\phi \Phi (\theta_{g})$$ (2)

In this equation, *N* denotes the number of images processed simultaneously and *ϕ*>0 serves as a weighting factor for constraint Φ(*θ*).

Although the source domain model enhances model generalization by extracting style-invariant features, it cannot guarantee the presence of effective semantic information in the features extracted by the encoder g. Hence, ISS generates random combinations of raw patches and augmented patches while preserving the order of the original patches.


$${ℒ}_{rec}(\theta )=\mathrm{argmin}\frac{1}{NK}{\sum_{n=1}^{N}\sum_{k=1}^{K}\left\{{\left\|p_{k}^{nm}-r(\tilde{f_{k}^{n}};\theta_{r})\right\|}_{2}^{2}\right\}}_{m=1}^{M}$$ (3)

In the equation, *M* denotes the random combinations’ number, *m* stands for the *m*_th_ random combination, *r* denotes the image reconstruction sub-network, $\tilde{f_{k}^{n}}\in \{f_{k}^{n},{\hat{f}}_{k}^{n},f_{bg}\}$ takes a probability of 1/3 for each value, and *f*_*bg*_ represents the features of patches with black backgrounds.

#### 3.2.2 Inter-image transfer

According to previous work [22], low-order feature statistics have domain specificity due to differences in distribution representation. Moreover, using *μ* and *σ* as style factors for semantic segmentation has been proven feasible in previous work [23]. Given image pairs (*x*_*a*_,*x*_*b*_), ISS randomly selects patches from *P*^*a*^ and *P*^*b*^ for all positions. The selected patches are arranged in the original order, i.e., $\tilde{P^{ab}}=[\tilde{P_{1}^{ab}},\tilde{P_{2}^{ab}},\cdots ,\tilde{P_{K}^{ab}}]$, where $\tilde{P^{ab}}\in (P_{i}^{a}\;P_{i}^{b})$ is randomly sampled with a probability of 1/2. Each image pair (*x*_*a*_,*x*_*b*_) is randomly selected from the current batch of images, and *x*_*a*_≠*x*_*b*_. Since there are inherent style differences between medical images, no additional image augmentation is required for inter-image generalization. Based on this, encoder g can distill patch features $\tilde{f^{ab}}=[\tilde{f_{1}^{ab}},\tilde{f_{2}^{ab}},\cdots ,\tilde{f_{K}^{ab}}]$. Subsequently, style factors *μ* and *σ* can separately calculate, and then style factors are exchanged and normalized between these features. The exchange process and normalization can be expressed as follows:

$${\ddot{f}}_{i}^{ab}=\sigma_{j}(\frac{\tilde{f_{i}^{ab}}-\mu_{i}}{\sigma_{i}})+\mu_{j},i,j\in [1,K]\wedge i\neq j$$ (5)

where *μ*_*j*_ and *σ*_*j*_ are the style factors of patch features $\tilde{f_{i}^{ab}}$, and ${\ddot{f}}_{i}^{ab}$ represents the result after exchange and normalization. The next step involves acquiring *M* random feature combinations for each batch of images to facilitate subsequent image reconstruction. Here, Eq (3) is reformulated accordingly:

$${ℒ}_{rec}^{'}(\theta )=\mathrm{argmin}\frac{1}{MK}{\sum_{k=1}^{K}\left\{{\left\|\tilde{p_{k}^{abm}}-r(\tilde{f_{k}^{abm}};\theta_{r})\right\|}_{2}^{2}\right\}}_{m=1}^{M}$$ (6)

where *a* and *b* represent the indices of image pairs randomly selected from [1, *N*], where the indices follow *a*≠*b*.

Based on this, the optimization objective function for inter-image transfer is expressed by:

$${ℒ}_{\mathrm{inter}}={ℒ}_{rec}^{'}$$ (7)

#### 3.2.3 Overall optimization objective of cross-patch style generalization and fine-tuning segmentation sub-network

The overall optimization objective of reducing the style sensitivity of the source domain model can be represented as the superposition of optimization objectives:

$${ℒ}_{cpsg}={ℒ}_{\mathrm{intra}}+{ℒ}_{\mathrm{inter}}$$ (8)

After reducing the style sensitivity through intra-image and inter-image transfer, a sub-network *s* requires training to facilitate medical image segmentation. Thus, the sub-network *s* undergoes fine-tuning on the source domain, incorporating original and augmented features, leveraging cross-entropy and Dice loss functions. Additionally, since it is expected that the encoder *g* dominates the entire fine-tuning process, the learning rate of *r* should significantly less than that of *g*. The optimization objective of the segmentation sub-network *s* is:

$${ℒ}_{seg}={ℒ}_{\mathrm{ce}}+{ℒ}_{\mathrm{dice}}$$ (9)

### 3.3 Adaptive confidence regularization

After the pre-training phase, SFDT-RSS initializes a new target model utilizing the pre-trained model. To further reduce the impact of style shift, we propose ACR loss to mitigate classification errors and address overfitting by employing dynamic scaling mechanism.

#### 3.3.1 Confidence maximization

The ACR loss reduces classification confusion by maximizing classification confidence. The correlation between any two classes *u* and *v* is defined as:

$$r_{uv}=\frac{tr({\hat{y}}_{u}{\hat{y}}_{v}^{T})}{HW}$$ (10)

where ${\hat{y}}_{u}\in {\mathbb{R}}^{HW\times 1}$ represents the probability of each pixel in the image belonging to class *u*. Correlation *r*_*uv*_ signifies the likelihood belonging to both *u* and *v*, and confidence could be estimated as:

$${ℛ}_{uv}=1-\frac{tr({\hat{y}}_{u}{\hat{y}}_{v}^{T})}{HW}$$ (11)

The logits center for *u* is computed by:

$${\bar{M}}_{u}=\frac{1}{HW}\sum_{i=1}^{HW}Z_{iu}$$ (12)

where *Z*_*iu*_ represents the center of logits for the *i*_th_ pixel output of class *u*. Based on this, the representation of adaptive weights is in the form of a diagonal matrix:

$$W_{u}=[\begin{array}{cccc} \left\|Z_{1u}-{\bar{M}}_{u}\right\| & & & \\ & \left\|Z_{2u}-{\bar{M}}_{u}\right\| & & \\ & & \ddots & \\ & & & \left\|Z_{(HW)u}-{\bar{M}}_{u}\right\| \end{array}]$$ (13)

Thus, the confidence described in Eq (11) can be re-expressed as:

$${ℛ}_{uv}^{w}=1-\frac{tr({\hat{y}}_{u},{\hat{y}}_{v}^{T}W_{u}W_{v})}{HW}$$ (14)

The objective of the ACR loss is to enhance discernibility, leading to clearer decision boundaries and less classification confusion:

$${ℒ}_{acr}=\frac{1}{C}\sum_{u=1}^{C}\sum_{v=1}^{C}\|1-{ℛ}_{uv}^{w}\|,\;u\neq v$$ (15)

Since high confidence predictions with errors only have a minor impact, Eq (15) can be selected as the confidence indicator to prevent error diffusion.

**Algorithm 1** SFDT-RSS workflow

**Data**: Source domain medical images: $D_{s}=\{X_{s}\in {\mathbb{R}}^{H\times W\times 1},Y_{s}\in {\mathbb{R}}^{H\times W\times C}\}$, target domain medical images: $D_{t}=\{X_{t}\in {\mathbb{R}}^{H\times W\times 1}\}$.

**Results**: Target domain model *M*_*t*_.

1. Pre-train source domain model *M*_*s*_;

2. Divide source domain medical images *x*_*s*_∈*X*_*s*_ into non-overlapping blocks;

3. Perform data augmentation on original blocks using random enhancement operator $A_{rand}(\cdot )$;

4. **while r**econstruction sub-network *r* not converged **do**

5. Extract features of original blocks $f_{k}^{n}$ and augmented blocks ${\hat{f}}_{k}^{n}$ via encoder *g*;

6 Enhance model’s generalization ability using ℒ_*cpsg*_ through intra-image and inter-image generalization;

7. **end**

8. Fine-tune segmentation sub-network using cross-entropy and Dice losses to obtain *M*_*s*_;

9. Adjust source domain model to obtain target domain model *M*_*t*_ (without using source domain data):

10. Initialize target domain model *M*_*s*_ with source domain model *M*_*t*_;

11. **while** Target domain model *M*_*t*_ not converged **do**

12. Calculate loss ℒ_*acr*_ based on model confidence and optimize model;

13. Employ dynamic scaling strategy to scale model output, avoiding overfitting;

14. **end**

**Output**: Converged target domain model *M*_*t*_.

#### 3.3.2 Dynamic scaling mechanism

Earlier research [24] has demonstrated method often exhibit overfitting to the operating domain, resulting in a tendency for model predictions to be uniformly distributed. In contrast to previous work [25] using fixed temperature coefficients for rescaling, the ACR loss adopts an adaptive regularization strategy. The scaling factor sets as 1 in the original training process, the dynamic regularization strategy does not take effect. When confidence is high, the impact of the scaling factor on model predictions can be ignored.


$${\hat{y}}_{iu}=\frac{\exp(Z_{iu}/\sum_{v\neq u}^{C}\left\|1-{ℛ}_{uv}^{w}\right\|)}{\sum_{u^{'}=1}^{C}\exp(Z_{iu^{'}}/\sum_{v\neq u^{'}}^{C}\left\|1-{ℛ}_{u^{'}v}^{w}\right\|)}$$ (16)

### 3.4 Algorithm summary

Through the training strategies outlined above, the problem of error diffusion in the source-free scenario can be effectively mitigated. This section outlines the training procedure of SFDT-RSS, which consists of two distinct stages: pre-training and domain transfer. The first phase focuses mainly on diminishing the dependency of the source domain model on a sole image style while bolstering the model’s capacity for generalization. The second phase is responsible for cross-domain knowledge transfer, further reducing the impact of style shift by reducing classification confusion. The procedure of SFDT-RSS is outlined in Algorithm 1.

In the pre-training phase, after data augmentation by the random enhancement operator $A_{\mathrm{rand}}(\cdot )$, deep features are extracted by the encoder g from the preprocessed source domain medical images. Based on the image reconstruction task and the reconstruction sub-network *r*, the model’s generalization ability is enhanced through intra-image and inter-image transfer mechanisms using ℒ_*cpsg*_. Then, the segmentation sub-network *s* is fine-tuned using a combination loss ℒ_*seg*_. This yields a source domain model with generalization capability across various stylistic contexts.

During the domain transfer phase, the target domain model is established by initializing it with the source domain model, after which this model is employed to predict segmentation on medical images from the target domain. Benefiting from the improved model generalization ability in pre-training, the predicted results of this model have high confidence. Then, based on these predicted results, the classification confidence is calculated, and the model is gradually corrected through optimization objective ℒ_*acr*_ to maximize this confidence, thereby enhancing discernibility. Simultaneously, a dynamic regularization mechanism is employed to adaptively scale the model’s predicted results, preventing overfitting and enhancing the final model’s segmentation performance.

## 4. Experimental validation

### 4.1 Dataset and preprocessing

To assess SFDT-RSS in the source-free cross-domain scenario for medical image segmentation, simulations and analyses were conducted on publicly available liver segmentation datasets LiTS [26] and Synapse [27], as well as retinal datasets REFUGE [28], Drishti-GS [29], and RIM-ONE-r3 [30]. The dataset information is summarized in Table 2.

**Table 2:** Information on the datasets used in this study.

Dataset	Collection site	Data annotation	Data type
LiTS [26]	Abdomen	Liver, Tumor	CT
Synapse [27]	Abdomen	Liver, Kidney, and 11 other organs	CT
REFUGE [28]	Fundus	Optic disc, Cup	RGB
Drishti-GS [29]	Fundus	Optic disc, Cup	RGB
RIM-ONE-r3 [30]	Fundus	Optic disc, Cup	RGB

The LiTS benchmark dataset includes 131 abdominal CT images, 107 of which contain lesions. This study focuses solely on liver segmentation. The 131 scans were sliced to obtain 2D images, resulting in a total of 58,638 images. Each image was resized to 224 × 224 pixels, with pixel values scaled to [0, 255] to obtain grayscale images for liver segmentation. The dataset information is summarized in Table 1.

The Synapse multi-organ segmentation dataset consists of 50 abdominal CT images, randomly selected from scans of ongoing colorectal cancer chemotherapy trials and abdominal hernia studies. In this study, 30 abdominal CT images were chosen. Each CT image comprised 85 to 198 slices of 224 × 224 pixels, resulting in a total of 3,779 abdominal clinical slices.

The REFUGE dataset contains 1,200 color fundus photographs (CFP) stored in JPEG format, each 8-bit per color channel, collected by ophthalmologists or technicians from upright seated patients. In this study, the original images were independently cropped multiple times and processed into 224 × 224 grayscale images, totaling 6,000 fundus images for optic disc and cup segmentation experiments.

The Drishti-GS dataset consists of retinal images manually annotated at the pixel level by multiple experts. In the released dataset, the fundus region was extracted by removing the non-fundus mask areas from the original images, resulting in fundus images with a resolution of 2047 × 1760 pixels. In this study, the dataset was processed similarly, and due to data limitations, it was used solely as the target domain.

The RIM-ONE-r3 dataset comprises retinal images, all captured under specific flash intensity settings using a Nidek AFC-210 fundus camera with a resolution of 21.1 megapixels. In this study, the dataset was used solely as the target domain.

### 4.2 Experimental design

To demonstrate the effectiveness of SFDT-RSS, this study compared it with three algorithms: the best existing supervised learning method, traditional transfer methods, and source-free transfer methods. The experiments encompassed liver segmentation as well as retinal optic disc and cup segmentation using the aforementioned datasets. Each experiment focused solely on the segmentation annotation of one category of pixels, i.e., binary image segmentation. The tasks LiTS → Synapse were used for liver segmentation validation, while tasks REFUGE → RIM-ONE-r3 and REFUGE → Drishti-GS were used for retinal optic disc and cup segmentation validation. The specifics of the three comparison methods are as follows:

Comparison with supervised learning methods: This aims to showcase the best results achievable by existing deep learning algorithms and analyze the performance loss in the source-free cross-domain scenario. During training, supervised models were trained by accessing both target domain data and their annotations. It’s worth noting that the source-free domain transfer model proposed in this paper only requires access to target domain data during training, without accessing source domain data or target domain annotations.

Comparison with traditional transfer methods: Traditional unsupervised domain transfer algorithms require access to source data and corresponding labels, and unlabeled target data during training. Comparing with such methods aims to evaluate model performance when source data is unavailable.

Comparison with source-free domain transfer Methods: The proposed SFDT-RSS belongs to this category of methods, where only the source domain model and target domain data can be utilized in the domain transfer phase, without accessing source domain data. Comparing with similar methods aims to more intuitively evaluate model performance.

### 4.3 Compared algorithms

To verify the effectiveness of SFDT-RSS in addressing the source-free cross-domain medical image segmentation problem, various studies in medical image segmentation were selected as benchmarks based on the three comparison paradigms described in the previous section.

#### 4.3.1 Supervised learning method

TransUNet [27]: Selected as a performance benchmark to analyze the performance loss caused by limiting factors in various scenarios.

#### 4.3.2 Traditional domain transfer method

AJTDA [31]: Compared to analyze the performance loss when source domain data cannot be accessed.

#### 4.3.3 Source-free domain transfer method

SFUDA [32]: Align the data distribution based on uncertainty and prior distribution perception strategies.

UBNA [33]: By incorporating an exponentially decaying momentum factor, the method adjusts a portion of the normalization layer statistics to match the target domain, thus facilitating model transfer.

SRDA [21]: Guides model transfer by using domain-invariant prior knowledge and minimizes the unlabeled entropy loss defined on target data.

DAE [34]: Adjusts target domain images through an image normalization submodule and explores implicit priors in prediction results, then models these implicit priors using independently trained denoising autoencoders.

DPL [20]: Utilizing two complementary denoising schemes at the pixel-level and class-level, which involve uncertainty and prototype estimation, respectively.

### 4.4 Analysis of experimental results

This section presents the relevant experimental results, including source-free cross-domain segmentation results of liver, optic disc, and cup in medical images, along with corresponding ablation experiments, computational complexity analysis, and parameter sensitivity analysis [35]. Given the relatively small dataset size, a lightweight ViT configuration was used in this experiment, with embedding dimension set to 192, embedding layers set to 4, and embedding heads set to 4. For the random augmentation operator $A_{\mathrm{rand}}(\cdot )$, the style randomization method from [36] was adopted, allowing an unlimited number of random style augmentation choices. The AdamW optimizer was used for model training with a learning rate of 1*e*−4 and a weight decay rate of 1*e*−3. The sensitivity of model parameters will be analyzed in detail in subsequent sections.

#### 4.4.1 Comparative analysis of source-free cross-domain segmentation experiment results

To assess the efficiency of SFDT-RSS for solving the source-free cross-domain medical image segmentation problem, this section presents the comparison results and discussions of our algorithm with supervised learning, traditional domain transfer, and source-free domain transfer methods. Firstly, the experiment precision of the proposed algorithm and comparison algorithms in terms of Dice coefficient and Average Surface Distance (ASD) for each task is reported, along with the standard deviation of experimental accuracy for each task. Then, the experimental results is provided. The segmentation accuracy of the model is positively correlated with the Dice coefficient and negatively correlated with the ASD metric. TransUNet, as the best existing supervised segmentation model, is used to demonstrate the upper bound of performance achievable by deep learning models on the corresponding segmentation tasks.

*(1) LiTS → Synapse*. Table 3 presents the accuracy comparison of liver segmentation on LiTS → Synapse task. A higher Dice coefficient and lower ASD indicate better segmentation performance. The Dice coefficient achieved by the supervised learning method TransUNet is 96.66%, with an ASD metric of 10.87, representing the highest performance achievable by existing deep learning models. The Dice coefficient of SFDT-RSS proposed in this paper is 1.84% lower than TransUNet, with an ASD metric 1.07 higher, indicating that SFDT-RSS achieves segmentation results close to the best existing supervised learning method in terms of both pixel overlap and contour similarity, effectively demonstrating the validity of our algorithm. Compared with traditional unsupervised domain transfer methods, SFDT-RSS is 0.40% lower in Dice coefficient than the AJTDA algorithm; for the ASD metric, SFDT-RSS is close to AJTDA, only 0.05 behind. This is mainly due to the inability of the proposed algorithm to access source domain data during training. Compared with source-free domain transfer methods, SFDT-RSS has significant performance advantages. In terms of the Dice coefficient, SFDT-RSS is 6.42%, 5.14%, 5.61%, 3.49%, and 2.83% higher than SFUDA, UBNA, SRDA, DAE, and DPL, respectively. For the ASD metric, SFDT-RSS is 1.31, 4.38, 2.82, 4.80, and 2.70 lower than existing methods, respectively, indicating that the proposed algorithm is superior to existing source-free domain transfer methods. This is because SFDT-RSS addresses the issue of error diffusion caused by style shift. Additionally, it can be observed that SFDT-RSS has a smaller standard deviation, indicating excellent stability of our algorithm.

**Table 3:** Performance comparison of all methods on the LiTS → Synapse task.

Method/Task		Liver segmentation	
		↑ Dice [%]	↓ ASD [pixel]
Supervised learning	TransUNet	96.66 ± 1.42	10.87 ± 6.57
Traditional domain transfer	AJTDA	95.22 ± 1.95	11.89 ± 5.52
Source-free domain transfer	SFUDA	88.40 ± 1.14	13.25 ± 8.89
	UBNA	89.68 ± 2.38	16.23 ± 8.04
	SRDA	89.21 ± 2.74	14.76 ± 12.61
	DAE	91.33 ± 3.51	16.74 ± 12.94
	DPL	91.99 ± 2.55	14.64 ± 9.10
	SFDT-RSS	94.82 ± 2.01	11.94 ± 8.18

*(2) REFUGE → RIM-ONE-r3*. Table 4 shows the accuracy comparison of optic disc and cup segmentation experiments on the REFUGE → RIM-ONE-r3 task. Similarly, TransUNet indicates the best performance achievable by deep learning models on the target domain dataset. Its Dice coefficient for optic disc segmentation task is 97.83% with an ASD metric of 8.63; for the cup segmentation task, the Dice coefficient is 88.60% with an ASD metric of 8.52. Compared with TransUNet, SFDT-RSS exhibits some gaps. This indicates that extracting source domain data distribution information and conducting knowledge transfer for this task in the source-free cross-domain scenario is quite challenging. However, from the table, it can be seen that, compared to existing traditional domain transfer and source-free domain transfer methods, SFDT-RSS still has significant performance advantages.

**Table 4:** Performance comparison on REFUGE → RIM-ONE-r3 task.

Method/Task		Optic disc segmentation		Cup segmentation	
		↑ Dice [%]	↓ASD [pixel]	↑ Dice [%]	↓ASD [pixel]
Supervised learning	TransUNet	97.83 ± 2.01	8.63 ± 3.84	88.60 ± 1.42	8.52 ± 4.79
Traditional domain transfer	AJTDA	92.76 ± 2.52	9.15 ± 4.12	83.36 ± 1.26	7.26 ± 4.55
Source-free domain transfer	SFUDA	84.47 ± 1.88	18.73 ± 6.05	72.49 ± 2.34	14.79 ± 9.53
	UBNA	84.53 ± 2.89	13.86 ± 5.65	74.54 ± 2.47	14.81 ± 8.99
	SRDA	87.44 ± 1.71	13.99 ± 2.69	76.91 ± 2.37	13.38 ± 6.75
	DAE	87.12 ± 1.41	11.78 ± 6.92	76.84 ± 2.47	13.82 ± 9.54
	DPL	88.79 ± 2.35	9.76 ± 3.76	77.78 ± 1.05	12.99 ± 5.67
	SFDT-RSS	91.43 ± 1.74	9.22 ± 3.44	80.99 ± 1.80	10.54 ± 4.46

Compared with traditional unsupervised domain transfer methods, for the optic disc segmentation task, the Dice coefficient of SFDT-RSS is 1.33% lower than the AJTDA algorithm, with an ASD metric 0.07 higher, indicating that the two have very similar segmentation results for the optic disc. For the cup segmentation task, the Dice coefficient of SFDT-RSS is 2.37% lower than the AJTDA algorithm, with an ASD metric 3.28 higher, indicating that the restriction of not being able to access source domain data in the source-free scenario has a greater negative impact on cup segmentation. This is mainly because cup images are greatly influenced by surrounding tissues, making them more difficult to segment than the optic disc. Compared with source-free domain transfer methods, the Dice coefficient of SFDT-RSS is 6.96%, 6.90%, 3.99%, 4.31%, and 2.64% higher than SFUDA, UBNA, SRDA, DAE, and DPL, respectively. For the ASD metric, SFDT-RSS is 9.51, 4.64, 4.77, 2.56, and 0.54 lower, respectively; for the cup segmentation task, the Dice coefficient of SFDT-RSS is 8.50%, 6.45%, 4.08%, 4.15%, and 3.21% higher than SFUDA, UBNA, SRDA, DAE, and DPL, respectively. For the ASD metric, SFDT-RSS is 4.25, 4.27, 2.84, 3.28, and 2.45 lower, respectively. These data indicate that SFDT-RSS has a significant advantage over existing source-free domain transfer methods for this task.

*(3) REFUGE → Drishti-GS task*. Table 5 presents the accuracy comparison of optic disc and cup segmentation experiments on the REFUGE → Drishti-GS task. TransUNet achieved Dice coefficients of 98.40% and 92.33%, with ASD metrics of 4.55 and 8.56 for optic disc and cup segmentation tasks, respectively. For the optic disc segmentation task, SFDT-RSS has a Dice coefficient 1.54% lower and an ASD metric 1.47 higher than TransUNet; for cup segmentation, its Dice coefficient is 6.37% lower and ASD metric 2.64 higher than TransUNet. These data indicate that SFDT-RSS performs close to supervised learning methods in this task without accessing source data, effectively demonstrating the validity of SFDT-RSS.

**Table 5:** Performance comparison of methods on REFUGE → Drishti-GS task.

Method/Task		Optic disc segmentation		Cup segmentation	
		↑ Dice [%]	↓ASD [pixel]	↑ Dice [%]	↓ASD [pixel]
Supervised learning	TransUNet	98.40 ± 0.84	4.55 ± 3.01	92.33 ± 1.09	8.56 ± 4.51
Traditional domain transfer	AJTDA	97.01 ± 0.88	4.22 ± 3.17	89.01 ± 1.55	10.64 ± 5.37
Source-free domain transfer	SFUDA	89.10 ± 2.36	6.01 ± 3.98	79.12 ± 2.79	15.70 ± 6.99
	UBNA	91.71 ± 1.64	9.88 ± 4.74	79.82 ± 2.68	15.67 ± 8.56
	SRDA	91.75 ± 1.37	8.67 ± 3.29	81.22 ± 1.15	14.84 ± 6.94
	DAE	92.86 ± 2.44	7.98 ± 4.87	82.38 ± 1.98	13.74 ± 7.58
	DPL	93.85 ± 1.68	7.14 ± 3.74	82.64 ± 1.67	12.17 ± 10.8
	SFDT-RSS	96.86 ± 1.03	6.02 ± 3.66	85.96 ± 0.97	11.20 ± 6.56

Compared with traditional unsupervised domain transfer methods, for the optic disc segmentation task, SFDT-RSS has a Dice coefficient 0.15% lower and an ASD metric 1.80 higher than the AJTDA algorithm; for the cup segmentation task, the Dice coefficient of SFDT-RSS is 3.05% lower and the ASD metric 0.57 higher than AJTDA. Similar to the REFUGE → RIM-ONE-r3 task, the results indicate that both methods have similar segmentation results for the optic disc, and the limitation of not being able to access source domain data in the source-free scenario has a greater negative impact on optic disc segmentation.

Compared with source-free domain transfer methods, SFDT-RSS exhibits the best performance in optic disc and cup segmentation. Compared to the suboptimal method DPL, SFDT-RSS has a Dice coefficient 3.01% higher and an ASD metric 1.12 lower in optic disc; in cup, the Dice coefficient is 3.32% higher and ASD metric 0.97 lower. Compared to other source-free domain transfer methods, SFDT-RSS has a greater performance advantage. Thus, SFDT-RSS outperforms existing source-free domain transfer methods on this task.

**Fig 3:**
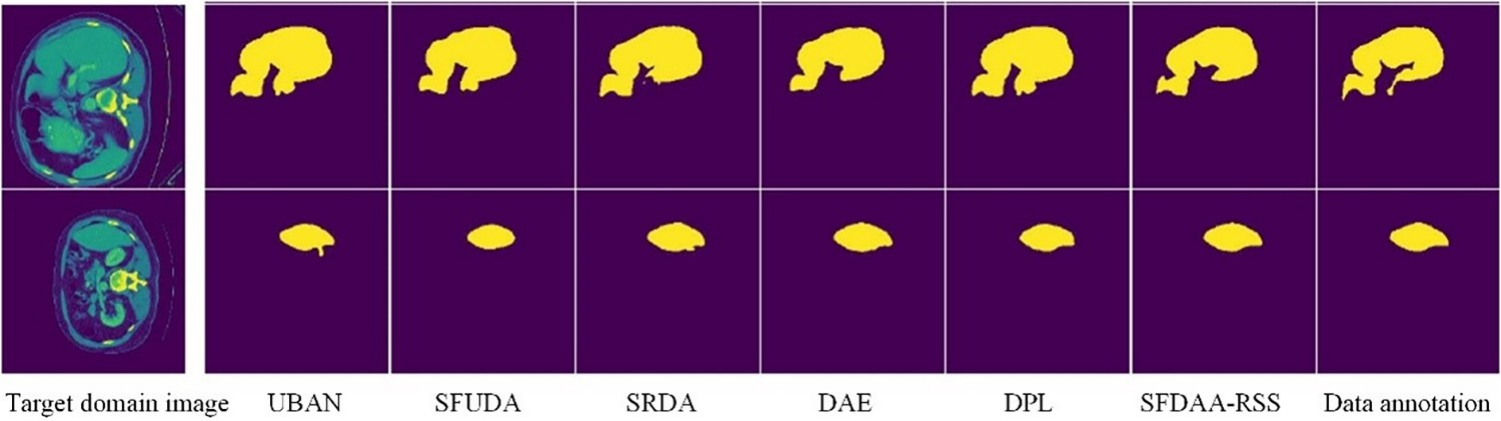
Results of liver segmentation task LiTS → Synapse

**Fig 4:**
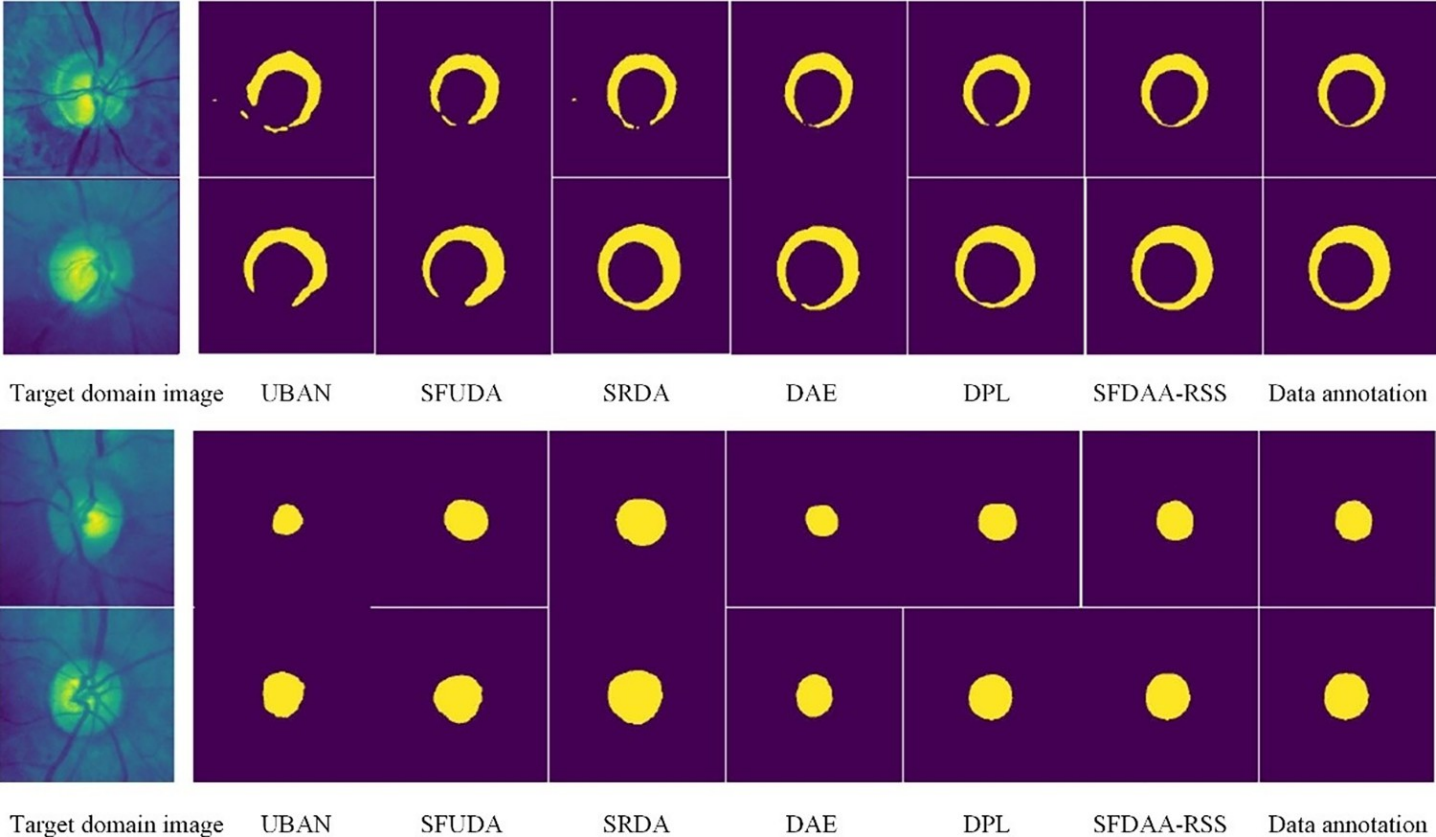
Results of Optic Disc (Top) and Cup (Bottom) Segmentation Task REFUGE → Drishti-GS.

*(4) Visualization results analysis*. Figs 3 and 4 respectively show the visual segmentation results of liver, optic disc (top), and cup (bottom), including a comparison of segmentation results between SFDT-RSS and existing source-free domain transfer methods. The liver segmentation result image is taken from the LiTS → Synapse task, while the optic disc and cup segmentation results are taken from the REFUGE → Drishti-GS task. The leftmost image represents the target domain test data, the rightmost image shows the corresponding pixel-level annotations, and the remaining images display segmentation results of various methods arranged from left to right in ascending order of segmentation accuracy. It can be observed from the figures that for the liver segmentation task, the segmentation effect of the proposed algorithm is significantly better in detail compared to other methods. For example, compared to the suboptimal method DPL, SFDT-RSS is more effective in segmenting image edges, with shapes closer to the original data annotations and no misclassification of pixels outside the liver. For the optic disc and cup segmentation tasks, the presence of large pixel value transitions in the original images poses great difficulties for accurate segmentation by the model, resulting in less-than-ideal segmentation results for all methods. However, compared to other methods, the segmentation results of SFDT-RSS are closer to the ground truth annotations, demonstrating the performance advantage.

#### 4.4.2 Sensitivity analysis

Ablation experiments are designed to evaluate SFDT-RSS, including comparisons of performance gains with the source-free strategy, the ISS module within SFDT-RSS, the ACR loss, and the performance gains with random masks. Additionally, as the ACR loss is model-agnostic, we combine it with other existing source-free domain transfer methods to further evaluate its effectiveness.

*(1) Performance gain analysis of the source-free domain transfer strategy*. To showcase the effectiveness of SFDT-RSS, we conduct a comparative analysis with baseline models across various tasks to elucidate the performance enhancements facilitated by the source-free strategy embedded in SFDT-RSS. The baseline model is derived from the ViT backbone network in SFDT-RSS, trained without the ISS mechanism and ACR loss during training, and then tested on the target domain. Compared with baseline model, the source-free domain transfer strategy in SFDT-RSS exhibits significant performance improvements across all tasks. For the LiTS → Synapse liver segmentation task, there is a 21.43% increase in Dice coefficient. For the REFUGE → RIM-ONE-r3 fundus segmentation task, there are improvements in optic disc segmentation Dice coefficient by 18.72% and cup segmentation Dice coefficient by 20.68%. Similarly, for the REFUGE → Drishti-GS fundus segmentation task, there are improvements in optic disc segmentation Dice coefficient by 20.44% and cup segmentation Dice coefficient by 19.57%. This is mainly attributed to the ISS mechanism in SFDT-RSS, which enhances the model’s generalization ability during source domain model training, reducing the sensitivity of the source domain model to image styles, thus improving the initial segmentation accuracy on the target domain medical images, to some extent, avoiding the problem of error diffusion. Based on this, SFDT-RSS adapts the source domain model to the target domain using the ACR loss, further addressing the problem of error diffusion by reducing the model’s tendency for misclassification, thereby enhancing the segmentation accuracy of medical images in source-free cross-domain scenarios.

*(2) Evaluation of the ISS module*. In this section, we verify the effectiveness of the cross-patch style generalization mechanism for liver segmentation tasks. As shown in Table 6, severe performance degradation occurs when SFDT-RSS does not use either intra-image or inter-image generalization. Specifically, without intra-image generalization, the Dice coefficient decreases by 1.64% and the ASD metric increases by 3.08. Similarly, without inter-image generalization, the Dice coefficient decreases by 1.39% and the ASD metric increases by 2.27. When both are disabled, the model’s performance loss is maximized, with a Dice coefficient decrease of 5.85% and an ASD metric increase of 9.37.

**Table 6:** Performance gain of the source strategy in SFDT-RSS (Dice).

Method/Task	LiTS→ Synapse	REFUGE → RIM-ONE-r3		REFUGE → Drishti-GS	
		Optic disc	Optic cup	Optic disc	Optic cup
Baseline model	73.39	81.24	60.31	76.42	66.39
SFDT-RSS	94.82	91.43	80.99	96.86	85.96
Performance gain	21.43	18.72	20.68	20.44	19.57

*(3) Evaluation of the ACR loss*. As shown in Table 7, not using the ACR loss when using the ISS mechanism results in performance degradation, with a Dice coefficient decrease of 1.50% and an ASD metric increase of 0.42, fully demonstrating the effectiveness of the ACR loss. As the ACR loss is model-agnostic, we further test its performance by adding it to other source-free domain transfer strategies. As shown in Table 6, adding the ACR loss to both supervised learning method TransUNet and source-free domain transfer method DPL results in varying degrees of performance improvement. Specifically, for the Dice coefficient, the performance improves by 1.05% and 0.85%, and for the ASD metric, it decreases by 1.85 and 1.62, respectively.

**Table 7:** Performance evaluation of framework modules for liver segmentation tasks.

ISS		ACR	RM	↑ Dice [%]	↓ ASD [pixel]
Intra-image	Inter-image				
		✓		88.97	21.31
✓		✓	✓	93.43	14.21
	✓	✓		93.18	15.02
✓	✓		✓	93.32	12.36
✓	✓	✓		93.64	14.06
✓	✓	✓	✓	94.82	11.94

*(4) Evaluation of random masking*. The random mask strategy is used solely for intra-image generalization by reconstructing masked images to enhance the model’s learning ability. As shown in Table 8, using random masking increases the Dice coefficient by approximately 1.2% compared to not using it. This performance gain mainly stems from the generalization effect obtained from the self-supervised image reconstruction task, fully demonstrating the effectiveness of random masking.

**Table 8:** Testing the performance improvement of ACR loss on other source-free strategies for liver segmentation tasks.

Method		TransUNet	DPL	SFDT-RSS
↑ Dice [%]	wo/ ACR	96.66 ± 1.42	91.99 ± 2.55	93.32 ± 2.23
	w/ ACR	97.71 ± 0.89	92.84 ± 2.09	94.82 ± 2.01
↓ ASD [pixel]	wo/ ACR	10.87 ± 6.57	14.64 ± 9.10	12.36 ± 9.02
	w/ ACR	9.02 ± 6.74	13.02 ± 8.75	11.94 ± 8.18

### 4.4.3 Sensitivity analysis of parameters

The impact of parameters M and K is discussed in the part. By exploring different values for these parameters, we aim to identify the optimal combination to ensure the model achieves the best segmentation performance.

**Fig 5:**
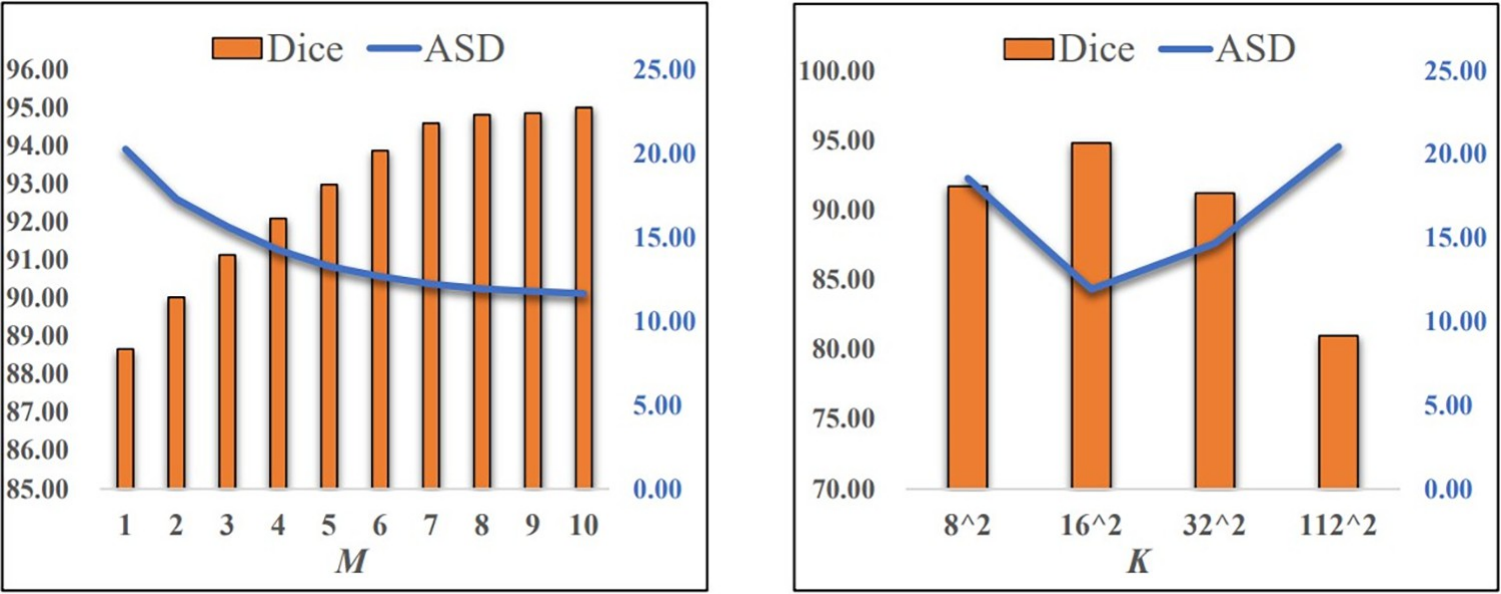
Sensitivity analysis of parameters M and K.

Parameter M controls the frequency of image reconstruction within the ISS module. Taking the liver segmentation task LiTS → Synapse as an example, we sequentially assign values from the set {1, 2,…, 10} to M, and the results for each value are depicted in Fig 5. It can be observed that, with K = 162, the Dice coefficient shows a positive correlation with M, while the ASD metric exhibits a negative correlation. As M increases, both the Dice coefficient and ASD metric tend to converge. However, due to the significant time consumption of the image reconstruction module during training and the limited performance improvement, we set M = 8 as the default value in our experiments to balance training costs and model performance.

Parameter K governs the quantity and size of blocks for medical image partitioning. To ensure each block has the same size, we sequentially assign values from the set {112^2^, 32^2^, 16^2^, 8^2^} to K. In other words, for an input image size of 224 × 224, each block corresponds to sizes {2^2^, 7^2^, 14^2^, 28^2^} respectively. The sensitivity of parameter K is visualized in Fig 5, showing that when K = 16^2^, the Dice coefficient reaches its maximum value, while the ASD metric achieves its minimum. This is because smaller block sizes are suitable for low-resolution images, whereas larger block sizes may lead to insufficient semantic representation. Hence, we adopt K = 16^2^ as the default setting to ensure optimal model performance.

## 5. Conclusions

This paper addresses the issue of error diffusion caused by style shift in existing unsupervised domain transfer algorithms for medical image segmentation, which occurs when knowledge transfer from the source domain data is not accessible. We propose a source-free domain transfer algorithm for medical image segmentation based on reducing style sensitivity. This algorithm enhances the generalization ability of the source domain model through a pre-training strategy, enabling the model to break free from reliance on a single image style. Subsequently, it employs an ACR strategy to reduce the model’s misclassification probability, further addressing the error diffusion issue and improving segmentation accuracy. Experimental results demonstrate that our proposed method, SFDT-RSS, achieves significant improvements in Dice coefficients for unsupervised cross-domain liver, optic disc, and optic cup segmentation tasks on five publicly available datasets: by 2.83%, 2.64%, 3.21%, 3.01%, and 3.32% respectively. These findings underscore the effectiveness of the SFDT-RSS in source-free cross-domain medical image segmentation tasks.
